# Digital Tool-Assisted Hospitalization Detection in the Tailored Antiplatelet Initiation to Lessen Outcomes due to Decreased Clopidogrel Response After Percutaneous Coronary Intervention Study Compared to Traditional Site-Coordinator Ascertainment: Intervention Study

**DOI:** 10.2196/47475

**Published:** 2023-11-10

**Authors:** Robert Avram, Julia Byrne, Derek So, Erin Iturriaga, Ryan Lennon, Vishakantha Murthy, Nancy Geller, Shaun Goodman, Charanjit Rihal, Yves Rosenberg, Kent Bailey, Michael Farkouh, Malcolm Bell, Charles Cagin, Ivan Chavez, Mohammad El-Hajjar, Wilson Ginete, Amir Lerman, Justin Levisay, Kevin Marzo, Tamim Nazif, Jean-Francois Tanguay, Mark Pletcher, Gregory M Marcus, Naveen L Pereira, Jeffrey Olgin

**Affiliations:** 1 University of California San Francisco San Francisco, CA United States; 2 Department of Medicine Montréal Heart Institute Université de Montreal Montréal, QC Canada; 3 Department of Cardiovascular Medicine Mayo Clinic Rochester, MN United States; 4 University of Ottawa Heart Institute Ottawa, ON Canada; 5 University of Ottawa Heart Institute Ottawa, MD United States; 6 National Heart, Lung, and Blood Institute National Institutes of Health Bethesda, MD United States; 7 St Michael's Hospital University of Toronto Toronto, ON Canada; 8 Cedars Sinai Health System Los Angeles, CA United States; 9 Mayo Clinic Health Systems La Crosse, WI United States; 10 Minneapolis Heart Institute Minneapolis, MN United States; 11 Albany Medical College Albany, NY United States; 12 Essentia Institute of Rural Health Duluth, MN United States; 13 NorthShore University Health System Evanston, IL United States; 14 Winthrop University Hospital Mineola, NY United States; 15 Columbia University Medical Center New York, NY United States; 16 Montreal Heart Institute Université de Montréal Montreal, QC Canada

**Keywords:** web-based digital study, randomized clinical trial, study app, clinical tracking cardiovascular hospitalizations, detection, digital tools, tool, clinical trial, research application, application, smartphone application, mobile phone survey, survey, hospital, accuracy, geofencing, cardiovascular, algorithm, hospital visit, mobile phone

## Abstract

**Background:**

Accurate, timely ascertainment of clinical end points, particularly hospitalizations, is crucial for clinical trials. The Tailored Antiplatelet Initiation to Lessen Outcomes Due to Decreased Clopidogrel Response after Percutaneous Coronary Intervention (TAILOR-PCI) Digital Study extended the main TAILOR-PCI trial's follow-up to 2 years, using a smartphone-based research app featuring geofencing-triggered surveys and routine monthly mobile phone surveys to detect cardiovascular (CV) hospitalizations. This pilot study compared these digital tools to conventional site-coordinator ascertainment of CV hospitalizations.

**Objective:**

The objectives were to evaluate geofencing-triggered notifications and routine monthly mobile phone surveys' performance in detecting CV hospitalizations compared to telephone visits and health record reviews by study coordinators at each site.

**Methods:**

US and Canadian participants from the TAILOR-PCI Digital Follow-Up Study were invited to download the Eureka Research Platform mobile app, opting in for location tracking using geofencing, triggering a smartphone-based survey if near a hospital for ≥4 hours. Participants were sent monthly notifications for CV hospitalization surveys.

**Results:**

From 85 participants who consented to the Digital Study, downloaded the mobile app, and had not previously completed their final follow-up visit, 73 (85.8%) initially opted in and consented to geofencing. There were 9 CV hospitalizations ascertained by study coordinators among 5 patients, whereas 8 out of 9 (88.9%) were detected by routine monthly hospitalization surveys. One CV hospitalization went undetected by the survey as it occurred within two weeks of the previous event, and the survey only allowed reporting of a single hospitalization. Among these, 3 were also detected by the geofencing algorithm, but 6 out of 9 (66.7%) were missed by geofencing: 1 occurred in a participant who never consented to geofencing, while 5 hospitalizations occurred among participants who had subsequently turned off geofencing prior to their hospitalization. Geofencing-detected hospitalizations were ascertained within a median of 2 (IQR 1-3) days, monthly surveys within 11 (IQR 6.5-25) days, and site coordinator methods within 38 (IQR 9-105) days. The geofencing algorithm triggered 245 notifications among 39 participants, with 128 (52.2%) from true hospital presence and 117 (47.8%) from nonhospital health care facility visits. Additional geofencing iterative improvements to reduce hospital misidentification were made to the algorithm at months 7 and 12, elevating the rate of true alerts from 35.4% (55 true alerts/155 total alerts before month 7) to 78.7% (59 true alerts/75 total alerts in months 7-12) and ultimately to 93.3% (14 true alerts/5 total alerts in months 13-21), respectively.

**Conclusions:**

The monthly digital survey detected most CV hospitalizations, while the geofencing survey enabled earlier detection but did not offer incremental value beyond traditional tools. Digital tools could potentially reduce the burden on study coordinators in ascertaining CV hospitalizations. The advantages of timely reporting via geofencing should be weighed against the issue of false notifications, which can be mitigated through algorithmic refinements.

## Introduction

Determining the occurrence of hospitalizations is crucial for the conduct of randomized clinical trials (RCT), as timely and accurate ascertainment of hospitalization end points are often key primary or secondary end points. However, standard methods used for ascertainment of these events can be laborious, requiring calls or study visits with all participants, including spending time contacting those participants (who are the majority of trial participants) who have not had a hospitalization [1]. Data collected via participant telephone calls endure recall bias [2], inefficiencies of finding mutual availability (“phone tag”), and reliance on review of electronic medical records that are site-specific and may miss detection of hospitalizations outside of the hospital network. This laborious process drastically increases the costs to conduct an RCT and in the era of pragmatic trials, cost-effective methods for capturing hospitalizations should be prioritized [3]. Recently, the importance of remote digital study follow-up was underscored by the COVID-19 pandemic, during which many conventional RCTs that required in-person recruitment and follow-up were suspended [4]. Internet-connected smartphones are ubiquitous and have the ability to precisely track the location of a participant and map it to real-world places, such as hospitals, allowing the follow-up of patients. Previously, remote RCT follow-up with passive detection of hospitalizations using smartphone GPS technology has been shown to be feasible [5]. Digital approaches, such as GPS tracking or self-reporting to ascertain hospitalizations could lessen coordinator burden and improve study efficiency, by streamlining workflow to allow site coordinators to focus attention only on participants with a digitally reported hospitalization and follow-up of participants who do not use these tools. Whether digital technologies can be used successfully in RCTs to detect hospitalizations when compared to traditional methods is not known.

The Tailored Antiplatelet Initiation to Lessen Outcomes Due to Decreased Clopidogrel Response after Percutaneous Coronary Intervention (TAILOR-PCI; NCT#01742117) trial was a large multicenter international RCT comparing point-of-care genotype-guided P2Y12 inhibitor therapy to conventional clopidogrel therapy. Initially, a 1-year of follow-up was planned [6]. The TAILOR-PCI Digital Study tested the feasibility of extending follow-up to 2 years using digital platforms and a low-contact approach (mailing letters and making phone calls rather than requiring clinic visits) for enrollment and engagement [7]. In this analysis, we took advantage of parallel ascertainment strategies (both a digital and traditional approach to ascertainment) of cardiovascular (CV) hospitalizations to evaluate the performance of digital strategies compared to the gold standard of study site coordinator follow-up and manual electronic health record (EHR) review.

## Methods

### Study Population

TAILOR-PCI began enrolling participants on May 29, 2013, finished enrollment on October 31, 2018, and completed the final planned study follow-up 1 year later, on October 31, 2019 [6]. The TAILOR-PCI Extended Follow-Up Study was designed to follow the RCT participants beyond the first year after randomization, for at least an additional year, to determine whether CYP2C19 genotyping could identify a group of participants that would benefit from extended dual antiplatelet therapy. In parallel, the TAILOR-PCI Digital Study was an ancillary study of the extended follow-up and enrolled participants in Canada and the United States, using a smartphone app with optional location tracking using a geofencing app to test the performance of digital tools to ascertain CV hospitalizations.

The design of the TAILOR-PCI Digital Study has been previously described [8]. Recruitment letters for the Digital Study were sent starting in February 2019. The Digital Study was built and conducted using the Eureka Research Platform [9], a direct-to-participant digital research platform [10]. TAILOR-PCI participants were enrolled in the Digital Study from 24 participating sites in the United States and Canada if they were within 24 months of initial randomization and had an Apple smartphone (Apple Inc) or an Android smartphone (Alphabet Inc). The follow-up of the Digital Study was completed on October 3, 2020.

### Recruitment

Recruitment was initiated by local TAILOR-PCI site study coordinators, who mailed letters to eligible participants inviting them to participate. Participants were instructed to visit the study website to learn more about the Digital Study, read, and sign the consent (if they chose to participate). After participants provided written informed consent, they received an SMS text with a link to download the study smartphone app and login. Those who did not consent after receiving the initial invitation letter were contacted via telephone by site study coordinators to ask them to participate, on up to 3 separate occasions. Participants could contact the clinical coordinating center for assistance downloading or using the study app.

### Oversight

Mayo Clinic (Rochester, Minnesota) was the clinical coordinating center for all participating sites and the University of California, San Francisco (UCSF) was the digital data coordinating center. The UCSF investigators developed the Eureka Research Platform smartphone app for the TAILOR-PCI Digital Follow-Up Study including the Eureka geofencing algorithm used in this study to map participant coordinates with hospital locations [5]. They also monitored the occurrence of geolocation hospitalization events and provided technical support to the clinical coordinating center throughout the study period. Mayo Clinic investigators conceived the study, received institutional review board (IRB) approval, and operationalized the implementation of the Digital Study, whereas UCSF investigators received institutional review board approval for the Eureka Research Platform and for serving as a digital data coordinating center. Each participating, eligible TAILOR-PCI site obtained local IRB approval for the study invitation material and for making participant contact. An independent National Heart, Lung, and Blood Institute–appointed Observational Study Monitoring Board was responsible for overseeing the conduct, safety, and data of the study.

### Ethical Considerations

The methods were performed in accordance with relevant guidelines and regulations. This study was approved by the Mayo Clinic IRB (11-006837). The Eureka Platform used to conduct this study was approved by the UCSF IRB (17-21879).

### Digital Ascertainment of Hospitalizations

After participants downloaded the Eureka study app, they were prompted to optionally consent to the smartphone location services (geolocation; Figure S1 in Multimedia Appendix 1). Geolocation services periodically monitored the location of the smartphone and returned latitude or longitude coordinates using the GPS and cell phone tower triangulation. The Eureka Research Platform geofencing algorithm triggered simultaneously an app notification with a request to fill out a hospitalization survey if the participant’s location was near a hospital or health care facility for a duration of ≥4 hours. The algorithm worked in the background to collect location data, not requiring the study app to remain open and not requiring any active interaction, once permission was granted by the participant. The Eureka geofencing algorithm was developed to minimize the impact on data transfer and battery performance. As part of the algorithm, the app determines when the movement to a new location has occurred, at which point the latitude and longitude of the patient are obtained via GPS. Each location was mapped to actual places in real time, using a web service (Google Places API) that returned an array of place names and categories given a latitude or longitude (ie, “Hospital,” “Health,” “Restaurant”). To define a hospital, the study team had a predefined dictionary of keywords including place names and place categories. In month 7 of the study, to reduce the alerts for nonhospital health facilities (eg, gyms and pharmacies were sometimes categorized as health establishments by the web service), a second dictionary of excluded places that were falsely identified as “health” facilities (such as gyms, aesthetics locations, pharmacies, etc) were added, empirically, after reviewing the list of places that triggered notifications. To trigger a geofencing survey, a participant had to be at a hospital location (ie, containing at least one of the predefined place names or place categories and not containing any of the excluded place names or place categories) for a period of ≥4 hours. Once these conditions were met, participants received a notification on their smartphone and had up to a week to complete the geofencing-triggered hospitalization survey (Table S1 in Multimedia Appendix 1), after which the survey would become unavailable. Participants were free to turn on or off the geolocation algorithm at any time in the study, via their smartphone settings. In addition, participants had access to the clinical coordinating center telephone line to troubleshoot issues with the Digital Study including the geofencing algorithm and for assistance with enabling or disabling the geolocation feature.

In parallel, participants were notified to complete a monthly hospitalization survey (Figure S1A in Multimedia Appendix 1) containing similar questions as the geofencing-triggered survey (Figure S1B in Multimedia Appendix 1), independent of any geofencing-triggered event. When these became available (once monthly), the participant received a notification from the Eureka app to complete the survey. If participants did not complete their monthly hospitalization or geofencing survey within 24 hours, they received a weekly automated text message and app notifications reminding them to complete study activities. Both the monthly mobile phone and geofencing surveys asked the participants whether they had been hospitalized, if they had stayed overnight, the admission and discharge dates, and the reason of hospitalization (Figure S1 in Multimedia Appendix 1).

### Study Coordinator Ascertainment of Hospitalizations

As part of the TAILOR-PCI Extended Follow-Up Study, site study coordinators contacted enrolled participants at 18 months and 24 months after the index PCI to assess vital status and ascertain interim hospitalizations. At least 3 attempts (on different days) were made to contact the participants by phone. At the same time points, the coordinators reviewed the medical records of those participants to determine whether a hospitalization occurred within the local EHR system. The study coordinator recorded the occurrence of any CV hospitalization, reported by the participant and documented in the medical record in a Case Report Form. For any CV hospitalization, they were also responsible for obtaining the actual medical record documentation if not available within the site’s EHR and forwarded relevant medical information to the Clinical Coordinating Center for subsequent independent adjudication of events. The Clinical Events Adjudication Committee, the Data Coordinating Center, and study coordinators were blinded to the results of the digital data and thus did not use any of the data collected in Eureka to determine hospitalizations. However, any hospitalization that was found by digital tools and that was not reported by conventional means was investigated by the Clinical Coordinating Center, and then subsequently adjudicated by the adjudication committee at the end of the study.

### Data Analysis

**Figure 1:**
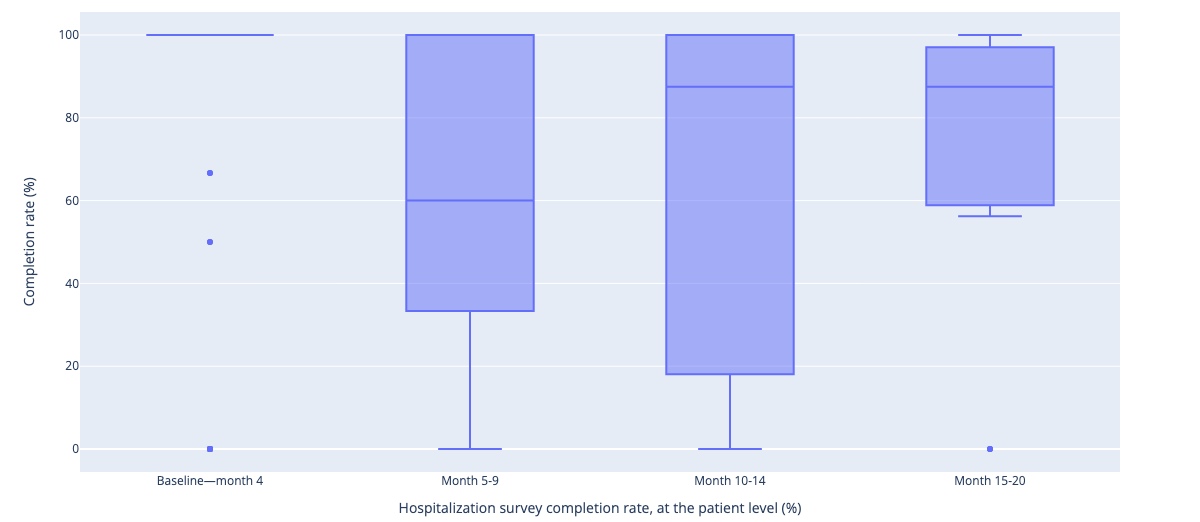
Median monthly hospitalization survey completion rate since the TAILOR-PCI: digital registry enrollment, at the participant level. TAILOR-PCI: Tailored Antiplatelet Initiation to Lessen Outcomes Due to Decreased Clopidogrel Response after Percutaneous Coronary Intervention.

Continuous variables are presented using mean and SD values if approximately symmetrically distributed and with median (IQR) reported otherwise and were compared using the *t* test or the Mann-Whitney test, as appropriate. Categorical variables are presented as frequencies (percentages) and compared using either chi-square or Fisher exact tests. The 2-tailed *P* values <.05 were considered statistically significant, without further correction for multiple testing. Binary outcomes are reported with 95% CI for the percentage, using the Agresti-Coull method for interval estimation [11]. To ensure the validity of our parametric statistical tests, we checked the distribution of our continuous variables for normality, using histograms and Q-Q plots, where a bell-shaped histogram or a Q-Q plot following the 45° line supported the assumption of normality. To further confirm these visual assessments, we used the Kolmogorov-Smirnov test, where a nonsignificant result (*P*>.05) from the Kolmogorov-Smirnov test supports the assumption that the data are normally distributed. CIs for continuous variables are estimated using normal approximations for the mean, using transformations as needed. First, we present the number of geofencing alerts (stratified by true alerts, meaning that they occurred when the participant was near or at a hospital, false alerts if they were triggered by a place that was a nonhospital health facility). In the figure, the number above the bars represents the percentage of participants that experienced false alerts and the dashed lines represent changes to the geofencing algorithm, done to reduce the rate of false positive alerts by eliminating nonhospital health facilities from geofencing. We present the performance of the digital methods of ascertainment of CV hospitalization using the site study coordinator ascertainment of these hospitalizations as the gold standard. We also present the completion rate of all methods of ascertainment of CV hospitalizations, as well as their respective true positive rate (number of confirmed CV hospitalizations per number of contact attempts). To do so, we present a statistical representation of the distribution of the completion rate of the monthly hospitalization survey at the participant level, through its quartiles. The ends of the box represent the lower and upper quartiles, while the median (second quartile) is marked by a line inside the box. Outlier data points are represented by a dot on the chart (Figure 1). We subsequently conducted a sensitivity analysis, truncating the follow-up period on March 1, 2020. This cutoff was chosen to assess our approach's performance in the pre–COVID-19 pandemic context, providing a clearer representation of the study's dynamics without the influence of the unprecedented global health crisis. Data were analyzed using Python (version 3.5; Python Software Foundation; using packages Panda’s version 1.2.4, scientific Python version 0.19.1, scikit learn version 0.19.0, and Plotly version 4.9.0), SPSS (version 10.0; IBM), and SAS (version 9.4; SAS Institute).

## Results

### Participation

There were 102 eligible participants (20 from Canada and 82 from the United States) who consented to the TAILOR-PCI Digital Study (out of 907 eligible patients from the United States and Canada in the Extended Follow-Up) with a median duration of follow-up of 6.9 (IQR 3.0-12.3) months. Among these, 17 participants did not download the Eureka mobile app (required for geofencing) but instead used their computers to participate in the Digital Study via the web. Therefore, there were 85 eligible participants who downloaded the study app and consented to the study, among whom 73 (85.8%) consented to geofencing. Participants who consented to the Digital Study and were eligible to consent to geofencing were older (64.7, SD 8.9 vs 62.2, SD 8.9; *P*=.02), Caucasian (90%, 92/102 vs 63% 2924/4645; *P*=.17), healthier, and with a higher prevalence of bachelor’s degree or higher educational level (Table S2 in Multimedia Appendix 1) compared to the rest of the Extended Follow-Up Study population. There was also a higher proportion of daily internet users (92%, 94/102 vs 50%, 1697/4645; *P*<.01). There was no difference in the geofencing consent rate between Canadian and American participants (70.7%, 58/82 vs 75.0%, 15/20; *P*=.70). The baseline characteristics of those participants that did not enable geofencing were like those who did, except for a lower incidence of family history of coronary artery disease and a lower prevalence of tablet use (Table 1). Participants that enabled geofencing had the algorithm turned on and transmitted data for 23.0 (IQR 6.5-82.0) days, which represented 28.1% (SD 33.8%) of each participant’s follow-up.

**Table 1:** Baseline characteristics.

Variable		Geofencing off (n=29)	Geofencing on (n=73)	*P* value	
**Age at randomization (years)**					.29
	Mean (SD)	63.2 (8.5)	65.3 (9.1)		
	Median (range)	64 (47-80)	65 (47-87)		
Male, n (%)		24 (83)	59 (81)	.82	
White, n (%)		25 (86)	67 (92)	.39	
**Country, n (%)**					.70
	Canada	5 (17)	15 (21)		
	United States	24 (83)	58 (79)		
**BMI, n (%)**					.20
	<25	4 (14)	12 (16)		
	25-30	10 (34)	35 (48)		
	>30	15 (52)	26 (36)		
Diabetes, n (%)		8 (28)	12 (16)	.20	
Hypertension, n (%)		19 (66)	49 (67)	.88	
Dyslipidemia, n (%)		21 (72)	52 (71)	.91	
Any history of heart failure, n (%)		0 (0)	2 (3)	.37	
Heart failure > 2 weeks, n (%)		0 (0)	1 (1)	.53	
Estimated Glomerular Filtration Rate using the Modification of Diet in Renal Disease equation <60, n (%)		4 (17)	9 (13)	.70	
Cigarette use, n (%)		2 (7)	4 (5)	.20	
History of myocardial infarction (excluding index event), n (%)		5 (17)	8 (11)	.39	
Peripheral artery disease, n (%)		0 (0)	3 (4)	.27	
History of Percutaneous Coronary Intervention, n (%)		7 (24)	19 (26)	.84	
History of coronary artery bypass graft, n (%)		1 (3)	10 (14)	.13	
Stroke or transient ischemic attack, n (%)		0 (0)	1 (1)	.53	
Family history of coronary artery disease, n (%)		10 (34)	45 (62)	.01	
Chronic lung disease, n (%)		0 (0)	4 (5)	.20	
Currently on dialysis, n (%)		0 (0)	0 (0)	N/A^a^	
**Education level, n (%)**					.90
	Less than high school	0 (0)	2 (3)		
	High school grad or some college	7 (25)	15 (21)		
	Associate or bachelor	13 (46)	35 (49)		
	Graduate or PhD	7 (25)	16 (23)		
	Prefer not to answer	1 (4)	3 (4)		
**Frequency of internet use, n (%)**					.34
	Does not use	1 (4)	1 (1)		
	About daily	24 (86)	67 (94)		
	About once a week	1 (4)	0 (0)		
	Occasionally (less than once a week)	2 (7)	2 (3)		
	Don't know	0 (0)	0 (0)		
	Prefer not to answer	0 (0)	1 (1)		
Has a computer or laptop, n (%)		26 (96)	62 (91)	.39	
Has a smartphone, n (%)		26 (100)	68 (99)	.54	
Has a tablet, n (%)		7 (29)	35 (56)	.03	
Has a smart-speaker, n (%)		10 (42)	16 (28)	.23	
Has downloaded app to phone, n (%)		25 (96)	65 (96)	.90	

^a^N/A: not applicable.

### Site Study Coordinator Ascertainment of Hospitalizations

All participants in the Digital Follow-Up Study were successfully contacted by the site coordinators at 12 months. In this population, study coordinators identified 9 overnight CV hospitalizations in 5 participants during the follow-up period using phone interviews and medical records searches. Coordinators successfully contacted all consented participants in the Digital Follow-Up Study at 24 months, with 171 phone calls, that found 9 potential health care contacts all of which were identified as CV hospitalizations after EHR review (“yield” of phone calls for CV hospitalization: 9/171 [5.2%]).

### Digital Ascertainment of Hospitalizations

In total, 8 of the 9 study coordinators ascertained hospitalizations described above were also detected by the participant-reported monthly digital hospitalization surveys (Table 2); 3 of these hospitalizations were also detected by the geofencing algorithm and confirmed by the triggered survey. About 6 hospitalizations, in 3 participants, were not detected by geofencing; 1 occurred in a participant who did not consent to geofencing (thus had geolocation turned off); and 5 occurred in 2 participants that had initially consented to geofencing but turned it off before the first hospitalization event occurred. Combining both the geofencing-triggered survey with the monthly hospitalization digital surveys (Tables S1 and S3 in Multimedia Appendix 1), the Digital Study detected 89% (8/9; 95% CI 54%-100%) of study coordinators detected CV hospitalizations. Hospitalizations detected by geofencing were “reported” by the participant within a median of 2 (IQR 1-3) days after the event, while those reported by monthly digital surveys were reported within a median of 11 (IQR 6.5-25) days after the event. In comparison, the site coordinator ascertained hospitalizations were obtained on the median, 38 (IQR 9-105) days after the event.

**Table 2:** Tailored Antiplatelet Initiation to Lessen Outcomes Due to Decreased Clopidogrel Response after Percutaneous Coronary Intervention hospitalizations reported by each method to the study coordinator^a^.

Hospitalization ID	Participant ID	Reported in monthly hospitalization survey	Detected by geofencing	Reported in the geofencing-triggered survey	Reported to the study coordinator
1	1	~✓^b^	~✓	~✓	✓
2	2	~✓	~✓	~✓	✓
3	2	~✓	~✓	~✓	✓
4	3	✓^c^	~^d^	~^d^	✓
5	4	✓	~^d^	~^d^	✓
6	5	✓	~^d^	~^d^	✓
7	5	Missed	~^d^	~^d^	✓
8	5	~✓	~^d^	~^d^	✓
9	5	~✓	~^d^	~^d^	✓

^d^This table includes only hospitalizations that were reported to the study coordinator. In the Digital Registry, we also had 3 participants report 3 additional noncardiovascular hospitalizations in the monthly survey, which were not reported to the study coordinator, and thus, impossible to adjudicate.

^b^~✓: Approximate date recorded by study coordinator.

^c^✓: Date recorded by study coordinator.

^d^~^:^ Geofencing turned off.

### Use of App-Based Monthly Hospitalization Digital Surveys

Participants completed a total of 69.0% (447/647 surveys, 95% CI 65.4%-72.5%) of all hospitalization-related monthly digital surveys available to them in the study app and 30 participants had at least a geofencing triggered survey and completed 73.3% (164/224; 95% CI 67.1%-78.6%) of them. Overall, the true positive rate (“yield”) of the monthly survey for CV hospitalizations was 8/447 (1.8%). Each participant completed a median of 100% (IQR 62.0%-100%; 46/85, 54.1% completed all surveys; 8/85, 9.4% completed none; and 31/85, 36.7% completed some but not all surveys) of their hospitalization surveys and 79% of their geofencing triggered surveys (IQR 50.0%-100%; 11/30, 36.7% completed all surveys; 19/30, 63.3% completed some surveys but not all). The average completion rate for the hospitalization survey shows variations across different time periods: 83.2% (84/101) during baseline to month 4, 67.4% (89/132) during months 5-9, 64.3% (137/213) during months 10-14, and 68.5% (137/200) during months 15-20. A series of pairwise chi-square tests reveal that the completion rate during “Baseline-month 4” is significantly higher compared to “Month 10-14,” “Month 15-20,” and “Month 5-9” (*P* value<.05). All participants who had a hospitalization event completed the monthly hospitalization survey that followed their discharge from the hospital, except for 1 patient, who had 2 hospitalizations 2 weeks apart, whereas they could only report 1 in the digital survey.

### Use of the App-Based Geofencing Algorithm

**Figure 2:**
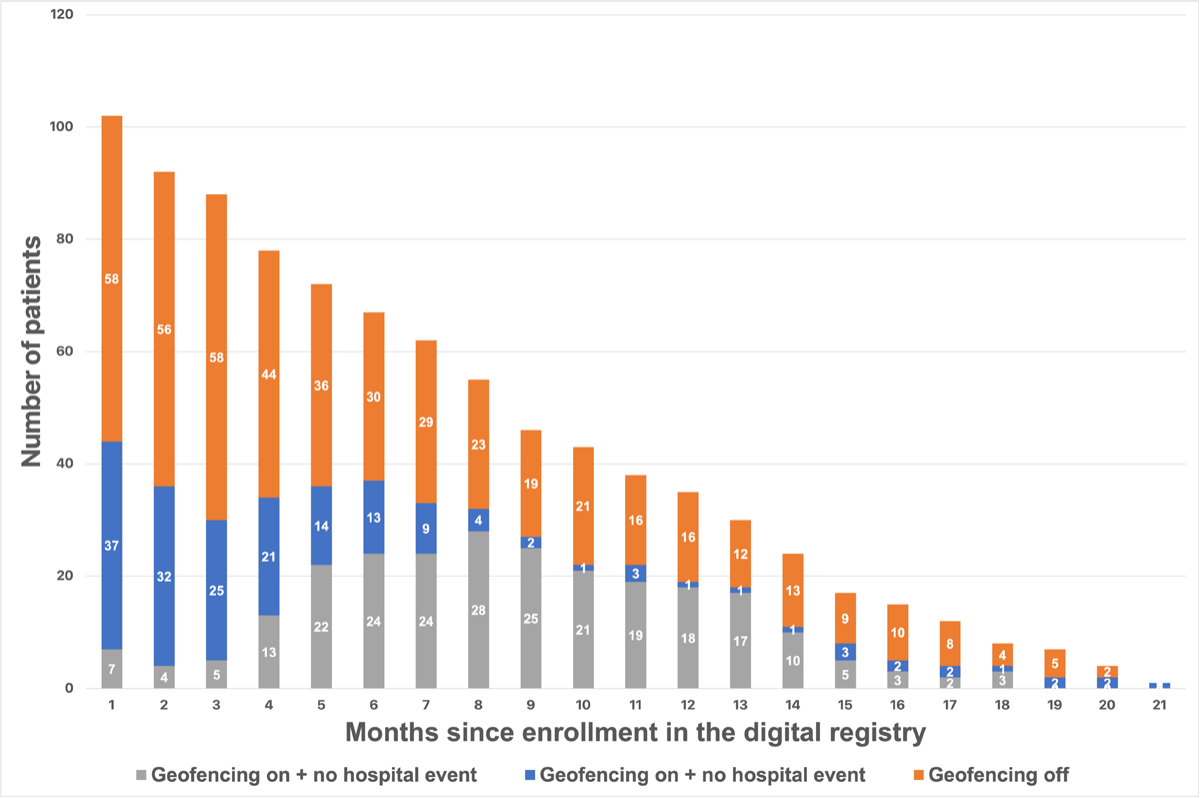
Consent to geofencing and number of positive alerts for potential hospitalization over time.

**Figure 3:**
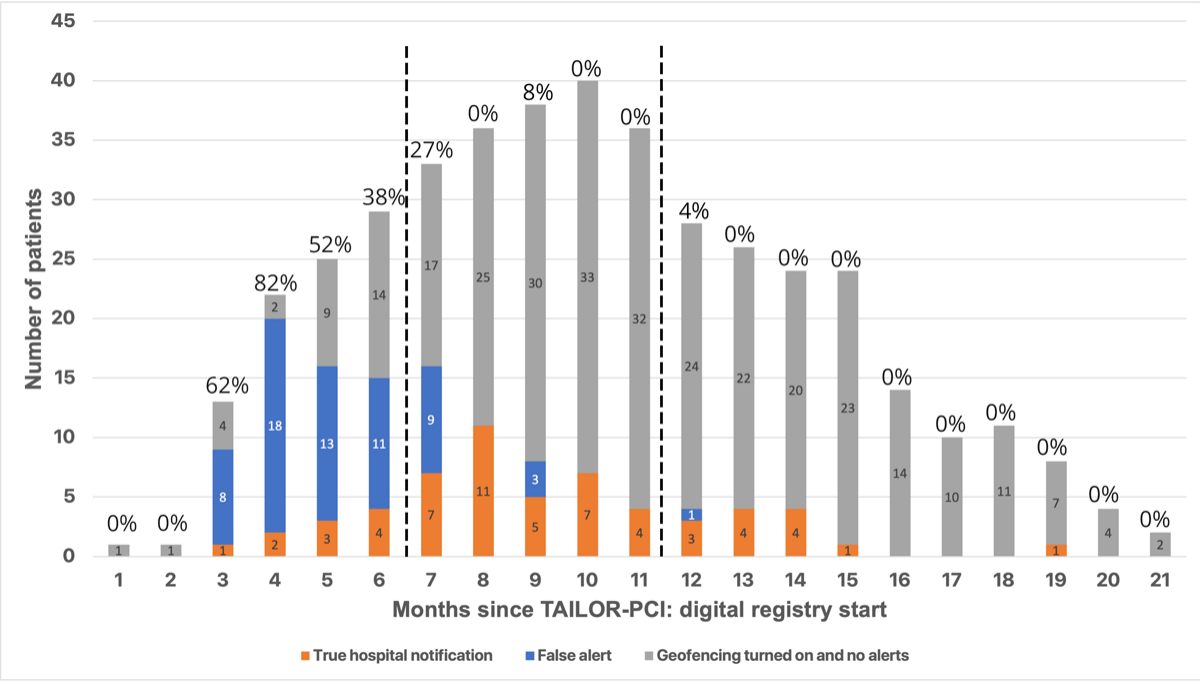
Number of participants with alerts according to the months since the TAILOR-PCI: digital registry start. The number above bars represents the percentage of participants that experienced false positive alerts. The dashed lines represent the changes to the geofencing algorithm, to reduce the rate of false positive alerts by eliminating nonhospital health facilities from geofencing. TAILOR-PCI: Tailored Antiplatelet Initiation to Lessen Outcomes Due to Decreased Clopidogrel Response after Percutaneous Coronary Intervention.

Throughout the study, the geofencing algorithm triggered 245 notifications in 39 participants (39/73, 53.4% of participants who consented to geofencing): 128 (52.2%, in 26 participants) were triggered by the true presence of the participant at a hospital, according to the mapping of the coordinates to the nearest real-world place, and 117 notifications (47.7%, in 30 participants) were due to labeling a participant’s location at a health facility but not at a hospital (“false notification’s;” Figure 2). After the study launch, the algorithm was improved iteratively, at month 7 and month 12 after initiation of the study, by adding keywords for exclusion from notifications representing names of places and place categories that were nonhospitals (eg, gyms, dentists, and aesthetic offices). This increased the rate of true alerts from 35.4% (55 true presence at hospital/155 notifications) prior to month 7, to 78.7% (59 true presence at a hospital in 75 notifications) between months 7 and 12, to 93.3% (14 true presence at a hospital in 15 notifications) after final adjustments were made at month 12 (Figure 3). Overall, 164 out of 245 (66.9%) instances of the geofencing-triggered hospitalization survey were completed, and 9 health care contacts were reported (5.5%, 9/164): 3 hospitalizations for CV causes (3/164, 1.8% “yield” for CV hospitalization of the geofencing survey) 2 hospital visits for CV causes, 2 hospitalizations for non-CV causes, and 2 hospital visits for non-CV causes. After adjustment to the algorithm at month 12, participants confirmed health care facility contacts in 5 out of the 14 instances (1 CV hospitalization, 2 CV visits, and 2 non-CV visits) and did not fill the geofencing survey in 9 instances. In our study, we included 20 participants from Canada and 82 from the United States. The comparative analysis between these 2 groups did not reveal any significant differences in terms of baseline characteristics, consent rates for geofencing, rates of geofencing event triggers, or detection of CV hospitalizations.

### Impact of the COVID-19 Pandemic

Among the 102 eligible participants, 39 continued their participation in the study beyond the onset of the COVID-19 pandemic. This implies that 63 participants completed the study prior to the pandemic's beginning. Prior to the pandemic, participants contributed geofencing data for an average of 52.5 (SD 62.6) days. This contribution significantly decreased after the pandemic onset to an average of 18.8 (SD 32.4) days (*P*=.02). Before the pandemic struck, 154 out of 230 (66.9%) geofencing-triggered hospitalization survey responses were recorded, and all instances of CV hospitalization happened during this timeframe. Once the COVID-19 pandemic began, only 1 participant had 15 separate geofencing events for repeated visits to the same hospital. This participant responded to the geofencing-triggered survey 10 times, each time reporting that they did not experience a CV hospitalization.

## Discussion

We used digital tools to ascertain CV hospitalizations in participants enrolled in the TAILOR-PCI Digital Study. Although the study was small, our key findings were (1) ascertainment of CV hospitalizations using a combination of monthly hospitalization surveys and geofencing-triggered surveys was feasible and correctly identified 8/9 (88.9%) instances of CV hospitalization when compared to the standard of study coordinator ascertainment with telephone follow-up and or EHR review; (2) the majority of Digital Follow-Up participants who consented to the digital study also consented to geofencing but subsequently removed access to geofencing; (3) after incorporating rules to not alert for nonhospital health facilities, the geofencing algorithm reduced the false positive alert rate 10-fold, from 64.6% (100 false alerts over 155 total alerts at month 7) to 6.7% (1 false alert over 15 total alerts between month 12-21); and (4) digital methods for reporting of CV hospitalization had a lower time latency than site coordinator ascertainment of events, resulting in documentation of CV hospitalization within days of the clinical event occurring, rather than weeks. Given the near ubiquitous use of smartphones and the importance of obtaining hospitalization data for research purposes, especially clinical trials, digital technology could provide a cost-effective method for collection and sharing outcomes in near real-time; this approach could then allow study coordinators to focus attention on a smaller group of participants who fail to use the digital tools or who report having a hospitalization (to collect details and records for adjudication), rather than concentrating their efforts on the entire study population, most of whom will not have hospitalizations to report.

The TAILOR-PCI Digital Study is one of the first studies to test the feasibility of extending the follow-up of an RCT using digital solutions [7]. Previously, the identification of health care contacts using smartphone geofencing technology had demonstrated a moderate sensitivity and positive predictive value of identifying hospital visits of ≥4 hours of 65% (102 out of 157 medical visits; 95% CI 57-72%) [5]. In the TAILOR-PCI study, the geofencing algorithm identified 128 health care contacts (out of 245 notifications; 52.2% yield), which is comparable with the previously published algorithm [1], but our approach has several advantages. The algorithm used in our study was a new version of the algorithm than that used by Nguyen et al [5], where, instead of mapping the coordinates to a predefined database of geofencing coordinates of eligible hospitals, we relied on an actively updated and dynamic database of places. Our solution worked across sites in the United States and in Canada, proving that it can be deployed across different health care systems, without requiring timely and expensive maintenance of a database of eligible hospitals. The early detection of CV hospitalizations through geofencing found in this study has important implications because it could allow for prompt therapeutic interventions by the study team and health care professionals, it would enable automatic and efficient patient monitoring and data collection and analysis to assess the effect of the intervention. This could, in turn, improve patient outcomes and experiences. Furthermore, using a list of keywords to define the hospital visits is flexible enough to eventually detect visits to myriad other kinds of places, such as fitness centers, liquor stores, or coffee shops, which could be used in other research settings. Such an approach was taken in the Coffee and Real-time Atrial and Ventricular Ectopy (CRAVE) study (NCT03671759) which targeted coffee shop visits to establish the link between arrhythmias and coffee drinking [12]. This tool is powerful enough to collect, in real time, visits to key places that could inform health-related behavior [13] and allow for near real-time intervention [14] for other diseases and not just CV hospitalization detection.

Despite these promises, the geofencing algorithm endured several limitations and a high rate of disabling this feature after the study started. Mobile phone operating systems currently allow for “turning off” access to location services independent of the app (and more recent operating systems make it more difficult to have geolocation running in the background), thus disabling geofencing, which affected the performance of this algorithm in our study. Some participants had the algorithm turned on and transmitted data for less than a third of their respective follow-ups. Additionally, it is important to note that post the onset of the COVID-19 pandemic, fewer participants contributed data, and they did so for a shorter duration. This may be attributable to the diminished mobility that came about with the introduction of lockdown measures during the early stage of the pandemic [15]. Essentially, the geofencing algorithm becomes inactive if no significant motion is detected, reflecting the reality of the decreased movement during this period. Moreover, although we did not observe this in our study, it is also possible that smartphones can be left at home and thus geofencing may not always be reliable for detecting hospitalizations. Therefore, to complement geofencing, a regularly scheduled survey (not reliant on geolocation), such as the monthly hospitalization digital surveys in our study, improved sensitivity to capture all except 1 instance of CV hospitalization. This missed CV hospitalization occurred less than 1 month after the previous CV hospitalization for the same patient, meaning that they could only report 1 of the 2 events since the survey occurs monthly. Future studies should allow the user to report a hospitalization as soon as it occurs, outside of a recurring survey, since sequential hospitalizations can occur more frequently than the scheduled survey.

Given the exponential increase in costs and complexity of conducting traditional RCTs [16,17], smartphone app could make clinical trial operations more cost-effective. Also, the majority of North Americans now use smartphones and participants are relying more on such devices to monitor their health [18,19], a trend that has accelerated since the onset of the COVID-19 pandemic, creating a ripe landscape for developing and integrating digital solutions for remote monitoring in research. Technological solutions, such as the Eureka Research Platform [10] can improve study design by including geofencing for real-time outcome capturing, digitally administered surveys that are scheduled on a predefined basis, and near real-time monitoring of participants using wearables [19]. In our current analysis, we observed a relatively low number of participants consenting to engage in the Digital Follow-Up Study. The reasons for this lower-than-expected enrollment have been previously discussed in depth [7]. Key factors contributing to this include the timing of the introduction of the digital component to the study, which was presented as an add-on rather than being integrated during initial recruitment. Additionally, our recruitment approach, which can be described as “low-touch,” relied primarily on mailed paper letters due to a lack of available email addresses for potential participants. Despite these shortcomings, we successfully integrated 2 of these tools and have described the consent rate and performance of these solutions. The traditional phone-based ascertainment of hospitalizations required 171 phone calls and had a 5.2% (9/171) yield but required significant work from the coordinators as they had to contact and survey the whole study population, the majority of whom were not hospitalized. Comparatively, the monthly survey and geofencing survey achieved a yield of 1.8% (8/447 for the monthly survey and 3/164 for the geofencing survey), respectively, for CV hospitalizations, but shifted the burden of data collection away from study coordinators. Therefore, in the digital tools, only 10 events (2 which were found to be non-CV hospitalizations) required review by the coordinators. Digital approaches for gathering hospitalization events could allow study coordinators to focus attention only on those participants who report a hospitalization or on those participants who fail to use the digital ascertainment tools (eg, those that have geolocation turned off and do not complete regular mobile phone surveys about hospitalizations), rather than in all participants in the study, as is currently done. Alternative methods for tracking hospitalizations could include leveraging databases such as the US National Death Index (NDI) [20] and the Canadian Discharge Abstract Database [21]. While these resources provide comprehensive and accurate data, their use may be limited by factors such as delays in data availability, complexities in data acquisition and handling, and an inability to track real-time patient movements or quickly detect hospitalizations, strengths that are inherent to geofencing methods. Nevertheless, the combination of such databases with geofencing could potentially enhance the robustness of hospitalization detection and provide a more holistic view of patient health outcomes.

Our study design achieved high engagement among consented participants for the monthly survey, but less so for the geofencing algorithm. Most participants completed all their monthly surveys but only a third of participants sent geofencing data for the duration of their study follow-up. This retention rate and continued participation in the study activities greatly exceed the median retention time of 5.6 days reported across digital studies [22]. This difference can be explained by the passive data collection of geofencing, requiring no participant interaction, as well as the automated and robust messaging reminding participants to complete missing activities weekly. Previous digital capture of end points did not use passive data collection methods and did not use an escalating reminder system, as is used in our platform [10] which was found to improve compliance in completing the study activities [16].

Our work offers valuable insights into the use of geofencing for ascertaining hospitalizations. First, evolving smartphone operating systems have become more privacy-centric and less permissive regarding passive data collection. Initially, location tracking required a single approval, allowing the algorithm to function in the background. However, with newer operating systems, participants needed to reapprove location tracking, leading to a higher dropout rate [23,24]. Moreover, indicators were added on the smartphone status bar to notify the participant that the location was being tracked. Repeated prompts also notified them of ongoing data collection and facilitated the disabling of geofencing leading to a high dropout rate for this intervention. Previous research has demonstrated that participants are willing to share confidential health-related information for research if their data are kept secure and confidential [25]. Although increased privacy and prevention of unwanted location tracking are beneficial, they hampered our study's monitoring ability. Future research should consider providing a framework for efficient location-data collection in a research setting, following informed consent and proper IRB oversight, while ensuring participants' privacy. For example, our geofencing algorithm within Eureka does not store all location data, only locations related to the primary outcome of the study, in this instance a hospital or health care facility, to protect the participant’s privacy.

Second, previous versions of geofencing algorithms were not optimized to minimize battery use. Our algorithm development has focused on using a battery-efficient approach in the hopes of maximizing compliance. Our geofencing algorithm activates GPS only when necessary (when sufficient movement and location changes were detected) and was designed to have minimal impact on battery life.

Third, 15% (17/102) of participants enrolled in the Digital Study were not comfortable with downloading the study app or enabling geofencing—which emphasizes the need for having another method for capturing hospitalizations (eg, regularly scheduled hospitalization surveys). The adoption of geofencing might also be improved with a high-touch enrollment and onboarding approach for reluctant participants. (eg, in-person with a study coordinator or using a call center) [16]. Furthermore, the initially observed false-positive detections and alerts could reduce study compliance over time. However, we rapidly decreased false-positive notifications by iteratively tuning the algorithm. Additionally, false alerts for participants who work or live near hospitals posed potential negative experiences. Implementing participant-specific algorithms with longer triggering times for surveys (eg, ≥24 h) could limit false alerts while potentially reducing sensitivity in detecting shorter visits such as emergency room evaluations. Moreover, it is important to note that the average age of our study population was 65 years. Experiences and adaptability to digital platforms might vary in older populations compared to younger cohorts. Differences can arise due to variations in technological familiarity and comfort, which could potentially reduce the efficacy of digital interventions. However, research has shown that older adults are more open to various technological or artificial intelligence applications within health care [26], and in our cohort, although not significant, patients who accepted geofencing were slightly older than those who did not. Future research should consider this demographic factor when designing digital health studies and interventions.

This study has several limitations. As previously reported, the population in the Digital Study, and those who consented to geofencing, were not fully representative of those enrolled in the main RCT due to factors such as higher education, healthier lifestyles, and greater technological literacy. Furthermore, we acknowledge that the smaller sample size and unique characteristics of the study participants, as compared to the broader pool of eligible patients, may have influenced the observed benefits of the study. Future research using randomization or well-matched control groups could yield more robust findings and minimize potential bias arising from baseline covariate discrepancies. We also did not observe differences between the US and Canadian participants in baseline characteristics, consent rates for geofencing or geofencing trigger rates, however, the generalizability of these results should be cautiously interpreted given the small cohort size of Canadian participants in the study. The digital study was an add-on and was not integrated during initial recruitment in the main RCT; incorporating such a digital study from the onset of the RCT would likely lead to a greater proportion of consented patients. Our study design precluded us from demonstrating any incremental benefit of the geofencing-triggered survey over the monthly hospitalization survey since both methods were activated concurrently. Consequently, all hospitalizations detected using geofencing were also self-reported via the monthly hospitalization survey. Future investigations should endeavor to ascertain the specific added value of geofencing over recurrent surveys and optimize the geofencing algorithm to ensure geolocation permissions remain active for the study's duration. In conclusion, our results suggest that combining monthly hospitalization surveys and geofencing-triggered surveys in an app-based follow-up approach may allow for more rapid ascertainment of most CV hospitalizations in the TAILOR-PCI Digital Registry than traditional study coordinator phone calls. These digital solutions could potentially enhance the efficiency of clinical trials, may offer reduced costs by reducing study coordinator effort and can reduce time latency in capturing CV hospitalizations compared to conventional methods. However, more research is needed to confirm these findings in larger and more representative populations. Our study showed regular usage of these digital tools by participants, which could substantiate its use for maintaining contact throughout the study follow-up duration. However, further research is necessary to fully understand and validate this technology in other settings.

## Appendix

Multimedia Appendix 1 Overview of the geofencing algorithm, geofencing-triggered and monthly hospitalization surveys. Comparison of participant characteristics between those who were eligible to consent to geofencing and those in the main TAILOR-PCI trial who were not.

## Abbreviations

CRAVE: Coffee and Real-time Atrial and Ventricular Ectopy
CV: cardiovascular
EHR: electronic health record
IRB: Institutional Review Board
NDI: US National Death Index
RCT: randomized clinical trials
TAILOR-PCI: Tailored Antiplatelet Initiation to Lessen Outcomes Due to Decreased Clopidogrel Response after Percutaneous Coronary Intervention
UCSF: University of California, San Francisco

## Data Availability

The data sets generated and analyzed during this study are not publicly available due to the inclusion of geofencing data, which if disclosed, could potentially compromise patient privacy but are available in a de-identified format from the corresponding author on reasonable request.
