# Supplementary material for: Digital Tool-Assisted Hospitalization Detection in the Tailored Antiplatelet Initiation to Lessen Outcomes due to Decreased Clopidogrel Response After Percutaneous Coronary Intervention Study Compared to Traditional Site-Coordinator Ascertainment: Intervention Study
Source: J Med Internet Res. 2023 Nov 10;25:e47475. doi: 10.2196/47475 (PMC10674150; doi:10.2196/47475)
Supplement: Multimedia Appendix 1 [file jmir_v25i1e47475_app1.pdf]

Supplementary Material

Digital Tool-Assisted Hospitalization Detection in the TAILOR-PCI Study Compared to Traditional Site-Coordinator Ascertainment: An Intervention Study Avram et al.

Supplementary Figure 1.

A. Geofencing Permission Prompt

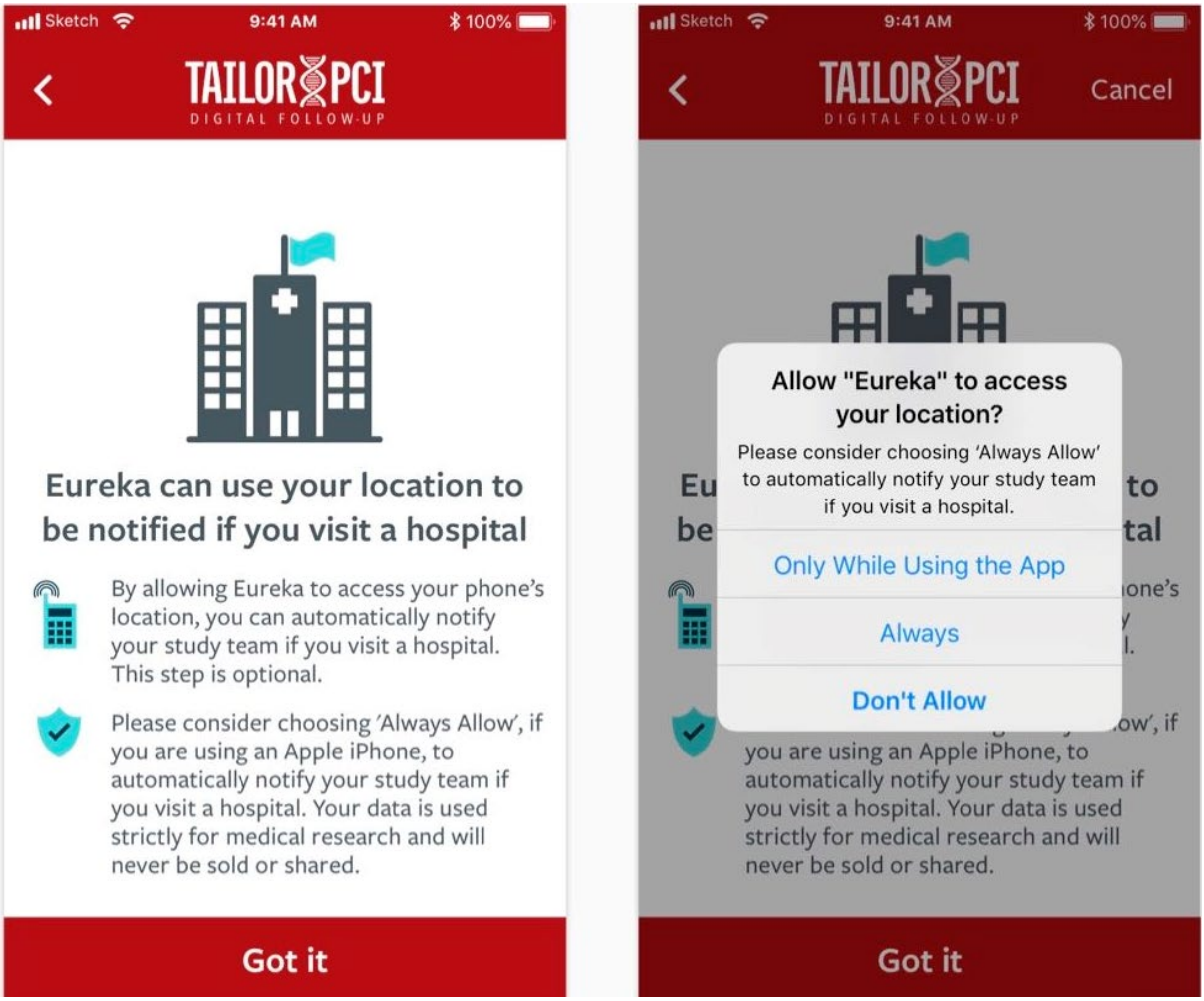

B. Geofencing Triggered Survey Prompt

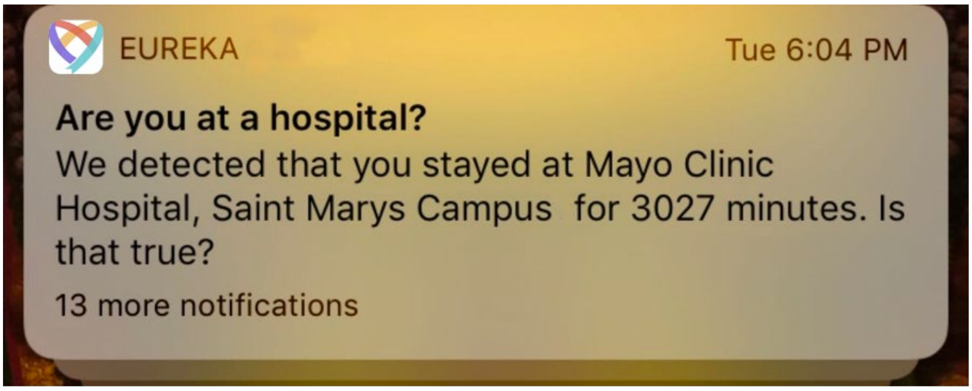

Supplementary Table 1. Geofencing-Triggered Hospitalization Survey

1. **Looks like you were near/at a hospital. Were you there for your medical care?**
    a. Yes
    b. No
2. **Did you stay at the hospital overnight? (triggered by answer to 1)**
    a. YES, I was hospitalized and I stayed overnight
    b. NO, I was discharged during the same day
    c. NO, I was an outparticipant
3. **When were you admitted at the hospital? (triggered by answer to 2)**
    a. Date
4. **When did you leave the hospital? (triggered by answer to 2)**
    a. Date
5. **Which hospital/clinic did you go to? (triggered by answer to 2)**
    a. City
    b. State/Province
    c. Hospital Name
6. **Was the main reason that you went to the emergency room or were admitted to the hospital related to your heart or heart condition? (You may want to look at the papers you received when you were discharged) (triggered by answer to 2)**
    a. Yes
    b. No
    c. I don't know
7. **What was the main reason for your hospitalization? (You may want to look at the papers you received when you were discharged) Pick all that apply. (triggered by answer to 2)**
    a. Heart attack, myocardial infarction (MI), STEMI or NSTEMI
    b. Angina or Chest Pain
    c. Heart Failure or pulmonary edema (fluid in the lungs)
    d. Stroke or Transient Ischemic Attack [TIA]
    e. Bleeding
    f. Arrhythmia (abnormal heart rhythm)
    g. Other
    h. I don't know
8. **Please describe any other diagnosis you had when you came to the hospital or emergency room. (triggered by answer to 2)**
    a. Other diagnosis

Supplementary Table 2.

Variable	TAILOR-PCI Extended Follow-Up participants<br>Refused to participate or not invited to the Digital Study - Not eligible for geofencing* (N=4645)	TAILOR-PCI Extended Follow-Up participants<br>Digital Study Participant - Eligible to consent to geofencing (N=102)	*P* Value
Age at randomization			0.019
Mean, SD	62.2 (11.0)	64.7 (8.9)	
Median (min, max)	62 (21, 95)	64 (47, 87)	
Male, n (%)	3506 (75%)	83 (81%)	0.17
Caucasian, n (%)	2924 (63%)	92 (90%)	<.001
Country, n (%)			<.001
Canada	1005 (22%)	20 (20%)	
Korea	1217 (26%)	0 (0%)	
Mexico	91 (2%)	0 (0%)	
USA	2332 (50%)	82 (80%)	
Body Mass Index, n (%)			0.06
<25	1230 (27%)	16 (16%)	
25-30	1746 (38%)	45 (44%)	
30+	1651 (36%)	41 (40%)	
Diabetes, n (%)	1264 (27%)	20 (20%)	0.09
Hypertension, n (%)	2908 (63%)	68 (67%)	0.40
Dyslipidemia, n (%)	2385 (51%)	73 (72%)	<.001
Any history of heart failure, n (%)	400 (9%)	2 (2%)	0.017
Heart failure > 2 weeks, n (%)	346 (7%)	1 (1%)	0.013
eGFR (MDRD)<60, n (%)	455 (11%)	13 (14%)	0.27
Cigarette Use, n (%)	1161 (25%)	6 (6%)	<.001
History of MI (excluding index event), n (%)	662 (14%)	13 (13%)	0.67
Peripheral Artery Disease, n (%)	116 (2%)	3 (3%)	0.78
History of PCI, n (%)	1068 (23%)	26 (25%)	0.55
History of CABG, n (%)	328 (7%)	11 (11%)	0.15
Stroke/TIA, n (%)	126 (3%)	1 (1%)	0.28
Family History of CAD, n (%)	1760 (38%)	55 (54%)	<.001
Chronic lung disease, n (%)	162 (3%)	4 (4%)	0.81
Currently on dialysis, n (%)	3 (0%)	0 (0%)	0.80

	TAILOR-PCI Extended Follow-Up participants		
**Variable**	**Refused to participate or not invited to the Digital Study - Not eligible for geofencing* (N=4645)**	**Digital Study Participant - Eligible to consent to geofencing (N=102)**	***P* Value**
Education level, n (%)			<.001
Less than HS	532 (16%)	2 (2%)	
HS grad/some college	1338 (39%)	22 (22%)	
Associate/Bachelor	932 (27%)	48 (48%)	
Graduate/PhD	309 (9%)	23 (23%)	
Prefer not to answer	286 (8%)	4 (4%)	
Frequency of internet use, n (%)			<.001
Does not use	837 (25%)	2 (2%)	
About daily	1697 (50%)	91 (92%)	
About once a week	251 (7%)	1 (1%)	
Occasionally (less than once a week)	252 (7%)	4 (4%)	
Don't know	141 (4%)	0 (0%)	
Prefer not to answer	219 (6%)	1 (1%)	
Which of the following do you have? A desktop, laptop,	1875 (82%)	88 (93%)	0.007
A smartphone (Android, iPhone, Blackberry, etc.), n (%)	1873 (82%)	94 (99%)	<.001
A tablet computer, n (%)	863 (39%)	42 (48%)	0.10
A smart-speaker (Alexa, GoogleHome, HomePod, etc.), n (%)	311 (15%)	26 (32%)	<.001
Ever downloaded an app to your phone?, n (%)	1503 (80%)	90 (96%)	0.007

Supplementary Table 3. Monthly Hospitalization Survey

1. **Were you hospitalized in the last month?**
    a. YES, I was hospitalized, and I stayed overnight
    b. NO, I was not hospitalized
2. **When were you admitted at the hospital? (triggered by answer to 1)**
    a. Date
3. **When did you leave the hospital? (triggered by answer to 1)**
    a. Date
4. **Which hospital/clinic did you go to? (triggered by answer to 1)**
    a. City
    b. State/Province
    c. Hospital Name
5. **Was the main reason that you went to the emergency room or were admitted to the hospital related to your heart or heart condition? (You may want to look at the papers you received when you were discharged.) (triggered by answer to 1)**
    a. Yes
    b. No
    c. I don't know
6. **What was the main reason for your hospitalization? (You may want to look at the papers you received when you were discharged) Pick all that apply. (triggered by answer to 1)**
    a. Heart attack, myocardial infarction (MI), STEMI or NSTEMI
    b. Angina or Chest Pain
    c. Heart Failure or pulmonary edema (fluid in the lungs)
    d. Stroke or Transient Ischemic Attack [TIA]
    e. Bleeding
    f. Arrhythmia (abnormal heart rhythm)
    g. Other
    h. I don't know
7. **Please describe any other diagnosis you had when you came to the hospital or emergency room. (triggered by answer to 1)**
    a. Other diagnosis
